# Dual regulation of lipid droplet-triacylglycerol metabolism and *ERG9* expression for improved β-carotene production in *Saccharomyces cerevisiae*

**DOI:** 10.1186/s12934-021-01723-y

**Published:** 2022-01-04

**Authors:** Xiao Bu, Jing‑Yuan Lin, Chang‑Qing Duan, Mattheos A. G. Koffas, Guo‑Liang Yan

**Affiliations:** 1 Centre for Viticulture and Enology, College of Food Science and Nutritional Engineering, China Agricultural University, Beijing, 100083 China; 2 Key Laboratory of Viticulture and Enology, Ministry of Agriculture and Rural Affairs, Beijing, 100083 China; 3 Innovation Research Center of Future Foods, College of Food Science and Nutritional Engineering, China Agricultural University, Beijing, 100083 China; 4 Key Laboratory of Food Bioengineering (China National Light Industry), China Agricultural University, Beijing, 100083 China; 5 Jiangsu Key Laboratory for High-Tech Research and Development of Veterinary Biopharmaceuticals, Jiangsu Agri-Animal Husbandry Vocational College, Taizhou, 225300 People’s Republic of China; 6 Center for Biotechnology and Interdisciplinary Studies and Department of Chemical and Biological Engineering, Rensselaer Polytechnic Institute, Troy, NY 12180 USA

**Keywords:** β-carotene, *Saccharomyces cerevisiae*, Lipid droplets, Oleic acid, *ERG9* down-regulation

## Abstract

**Background:**

The limitation of storage space, product cytotoxicity and the competition for precursor are the major challenges for efficiently overproducing carotenoid in engineered non-carotenogenic microorganisms. In this work, to improve β-carotene accumulation in *Saccharomyces cerevisiae*, a strategy that simultaneous increases cell storage capability and strengthens metabolic flux to carotenoid pathway was developed using exogenous oleic acid (OA) combined with metabolic engineering approaches.

**Results:**

The direct separation of lipid droplets (LDs), quantitative analysis and genes disruption trial indicated that LDs are major storage locations of β-carotene in *S. cerevisiae*. However, due to the competition for precursor between β-carotene and LDs-triacylglycerol biosynthesis, enlarging storage space by engineering LDs related genes has minor promotion on β-carotene accumulation. Adding 2 mM OA significantly improved LDs-triacylglycerol metabolism and resulted in 36.4% increase in β-carotene content. The transcriptome analysis was adopted to mine OA-repressible promoters and *IZH1* promoter was used to replace native *ERG9* promoter to dynamically down-regulate *ERG9* expression, which diverted the metabolic flux to β-carotene pathway and achieved additional 31.7% increase in β-carotene content without adversely affecting cell growth. By inducing an extra constitutive β-carotene synthesis pathway for further conversion precursor farnesol to β-carotene, the final strain produced 11.4 mg/g DCW and 142 mg/L of β-carotene, which is 107.3% and 49.5% increase respectively over the parent strain.

**Conclusions:**

This strategy can be applied in the overproduction of other heterogeneous FPP-derived hydrophobic compounds with similar synthesis and storage mechanisms in *S. cerevisiae*.

**Graphical Abstract:**

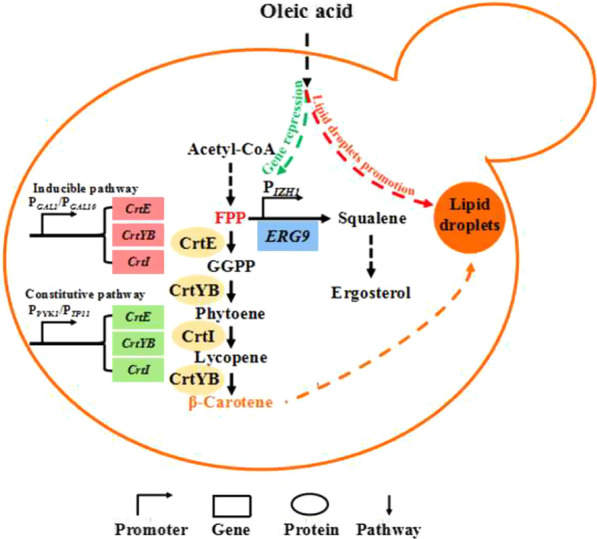

**Supplementary Information:**

The online version contains supplementary material available at 10.1186/s12934-021-01723-y.

## Background

Carotenoids are an important group of natural and liposoluble pigments with multiple physiological and nutritional functions, that are widely used as food colorants, food and cosmetics additives, health supplements, animal feeds and nutraceuticals [1]. Global market for carotenoids reached $1.5 billion in 2018 and is expected to grow to $2.0 billion by 2022 [2]. Although the demand for carotenoids is rapidly increasing, their supply is limited due to inefficient production methods. In recent years, with advances in metabolic engineering and synthetic biology, engineered non-carotenogenic microorganisms have become a primary alternative for economic and mass production of carotenoids [3–5]. Among which, the budding yeast *Saccharomyces cerevisiae* is a particularly attractive host due to its well characterized genetics, physiology, and metabolism, as well as the availability of diverse genetic toolkits for its engineering [6]. In addition, it is also GRAS (generally regarded as safe) and amenability to industrial bioprocess conditions.

Carotenoids are synthesized through the mevalonate (MVA) pathway in *S. cerevisiae*. Co-expression of the exogenous genes such as GGPP synthase (crtE), phytoene synthase (CrtB) and phytoene desaturase (CrtI), or bifunctional phytoene synthase and lycopene cyclase (crtYB) lead to metabolic flux towards heterogeneous pathway to synthesize lycopene and β-carotene [4]. To overproduce carotenoid products, various metabolic engineering approaches were deployed, such as screening and engineering exogenous enzymes with high activity [7, 8], engineering the MVA pathway and central carbon module to maximize precursor supply [9, 10], optimizing the cofactor supply (NADPH and ATP) and multi-module engineering for flux balance [11], and mining and engineering endogenous and exogenous transporters to efflux product [12, 13]. However, to date, carotenoid yields in engineered microorganisms are still low and do not meet industrial production requirements. Therefore, further strain improvement by metabolic engineering is required. Indeed, to achieve this goal, there are three major challenges that need to be addressed. Firstly, reduce the toxicity of product. The accumulation of heterogeneous carotenoids can cause cytotoxicity and introduce an undesirable metabolic burden, which adversely affect cell growth and product synthesis [14]. Additionally, due to depositing in the cellular membrane bilayer, carotenoid accumulation would destruct the membrane integrity and cause membrane stress [14, 15]. To address this issue, separating cell growth phase with product synthesis phase by dynamic control modules, or relieving the membrane stress by overexpressing unsaturated fatty acids (UFAs) genes have been used in the past [10, 15]. Secondly, enlarge the product storage space of the host organism. From cellular physiology point of view, it seems to be difficult to accumulate carotenoids to a high concentration in cells due to their toxicity. Fortunately, microbes have developed various strategies to store these toxic compounds. All carotenoids are stored in the cellular membrane of prokaryotic cells after synthesis, expanding the membrane surface area and thus facilitating carotenoid overproduction in *Escherichia coli* [16]. On the other hand, in yeast such as *S. cerevisiae,* carotenoids are believed to be stored in cellular membranes or (and) neutral lipid droplets (LDs), although the distribution proportion is still not clear. Up-regulating phospholipid and sterol biosynthesis [9], or improving LDs formation [17] could all lead to improvement of lycopene yield in *S. cerevisiae*. To our best knowledge, until now, no related work has been conducted on β-carotene accumulation in *S. cerevisiae.* Thirdly, rational balance of the competition for precursors such as farnesyl diphosphate (FPP) between native components and carotenoid synthesis, because the precursor not only serves as a building block for non-native isoprenoids, but also for essential cellular components, such as ergosterol. Minimizing metabolic fluxes towards side pathways and diverting them to heterologous metabolic reactions is a smart method [18]. In this regard, down-regulation of *ERG9* gene which encodes squalene synthase (the first committed step after farnesyl diphosphate in ergosterol biosynthesis), using the methionine-repressible *MET3* promoter [19], *HXT1* promoter [10] and ergosterol-responsive promoters [20] resulted in significant production improvements.

Considering that the boost effects of various strategies can be synergistic, it is believed that the highest improvement in yield could be achieved with multiple strategies implemented simultaneously. In the present work, we focused on the multi-function of oleic acid (OA), a cheap UFA. We developed a strategy that simultaneously increases cell storage capability, and strengthens metabolic flux to carotenoid pathway with relieving cell membrane stress by utilizing exogenous OA combined with metabolic engineering approaches. Firstly, we quantitatively proved LDs is the major storage location of β-carotene in *S. cerevisiae*, and optimized the concentration of OA promoting LDs formation, to expand the storage space of β-carotene. We then mined a series of OA-repressible promoters to replace *ERG9* through transcriptional assay and identified *IZH1* as a suitable promoter to dynamically down-regulate *ERG9* expression without adversely affecting cell growth. After introducing an extra constitutive β-carotene synthesis pathway to obtain sequential control β-carotene biosynthesis, the intracellular content and yield of β-carotene in final strain achieved 107.3% and 49.5% increment compared to the original strain, respectively.

## Results and discussion

### LDs are the major location for β-carotene storage in *S. cerevisiae*

**Fig. 1:**
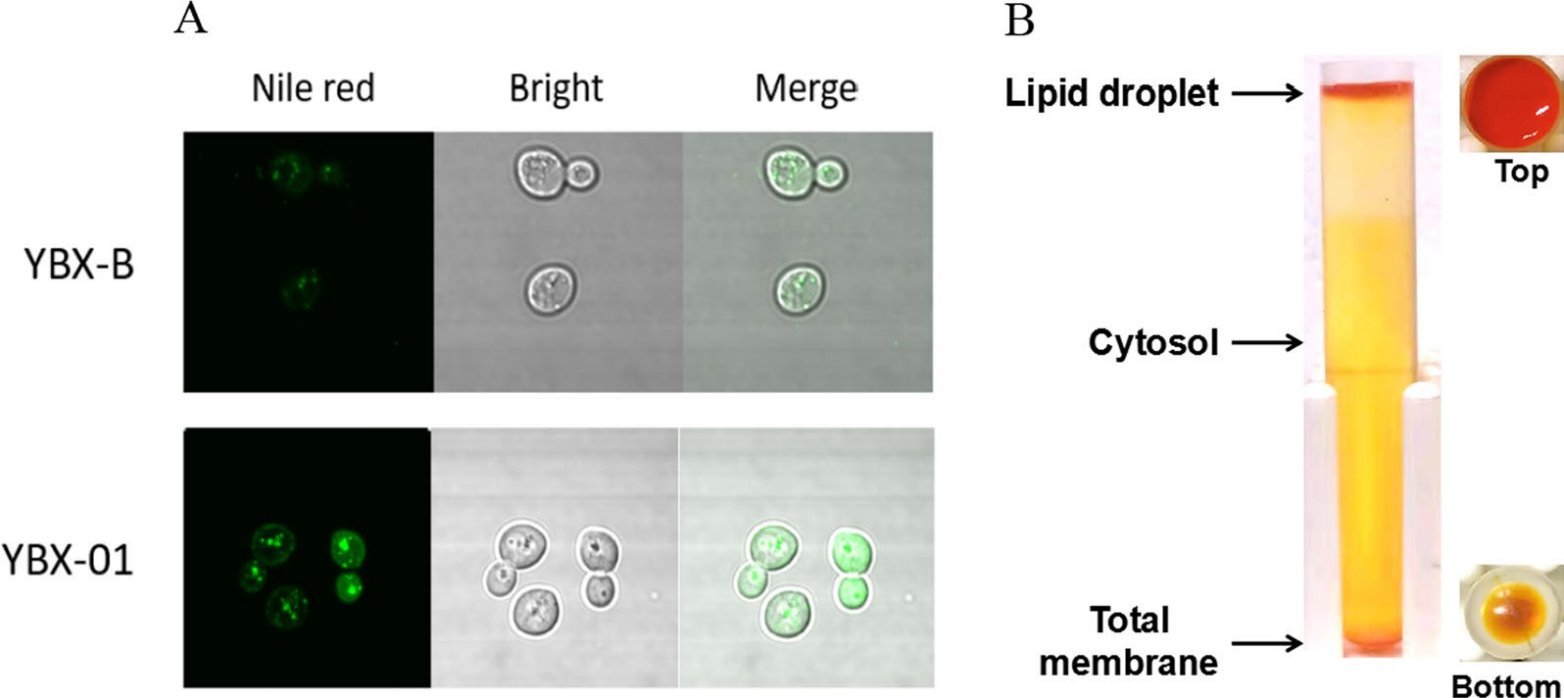
**A** Laser confocal images of β-carotene producing strain YBX-01 and the parent strain YBX-B stained with Nile red. The excitation wavelength of Nile red is 488 nm; Bright is white light; Merged is combined field of Nile red and Bright. **B** Separation of yeast lipid droplets by ultracentrifugation

To expand the storage space for improving β-carotene accumulation, it is vital to identify the major storage location of β-carotene in *S. cerevisiae*. At present, β-carotene is believed to be stored in cell membrane or (and) LDs, but the distribution ratio is not clear [9, 17]. Herein, we quantitatively determined this value. Firstly, we visualized the number of LDs in the wild-type strain YBX-B and β-carotene synthesizing strain YBX-01 (producing 5.50 mg/g DCW β-carotene) constructed in our previous work [12] by transmission electron microscope (Additional file 1: Fig. S1) and confocal microscope (Fig. 1A). The results all show that more LDs are produced in strain YBX-01, indicating that the budding yeast would produce extra LDs to respond to carotenoid accumulation. Then we directly separated LDs and cell membrane components for β-carotene quantitative determination, respectively. According to the previously reported protocol [21], the cells of strain YBX-01 cultured for 72 h were disrupted, and various components were collected based on the density difference. After ultracentrifugation of the post-nuclear supernatant (PNS) at 40,000 rpm for 1 h, LDs were enriched in the upper layer of the SW40 tube, which clearly indicated that a large amount of orange β-carotene exist in LDs (Fig. 1B). The cytoplasmic components were located in the middle layer, while the total membrane components were at the bottom of the centrifuge tube. The following quantitative analysis shows that LDs contained 85.5% of β-carotene (4.70 mg/g DCW), while the cell membrane only contained 9.4% (0.52 mg/g DCW).

**Fig. 2:**
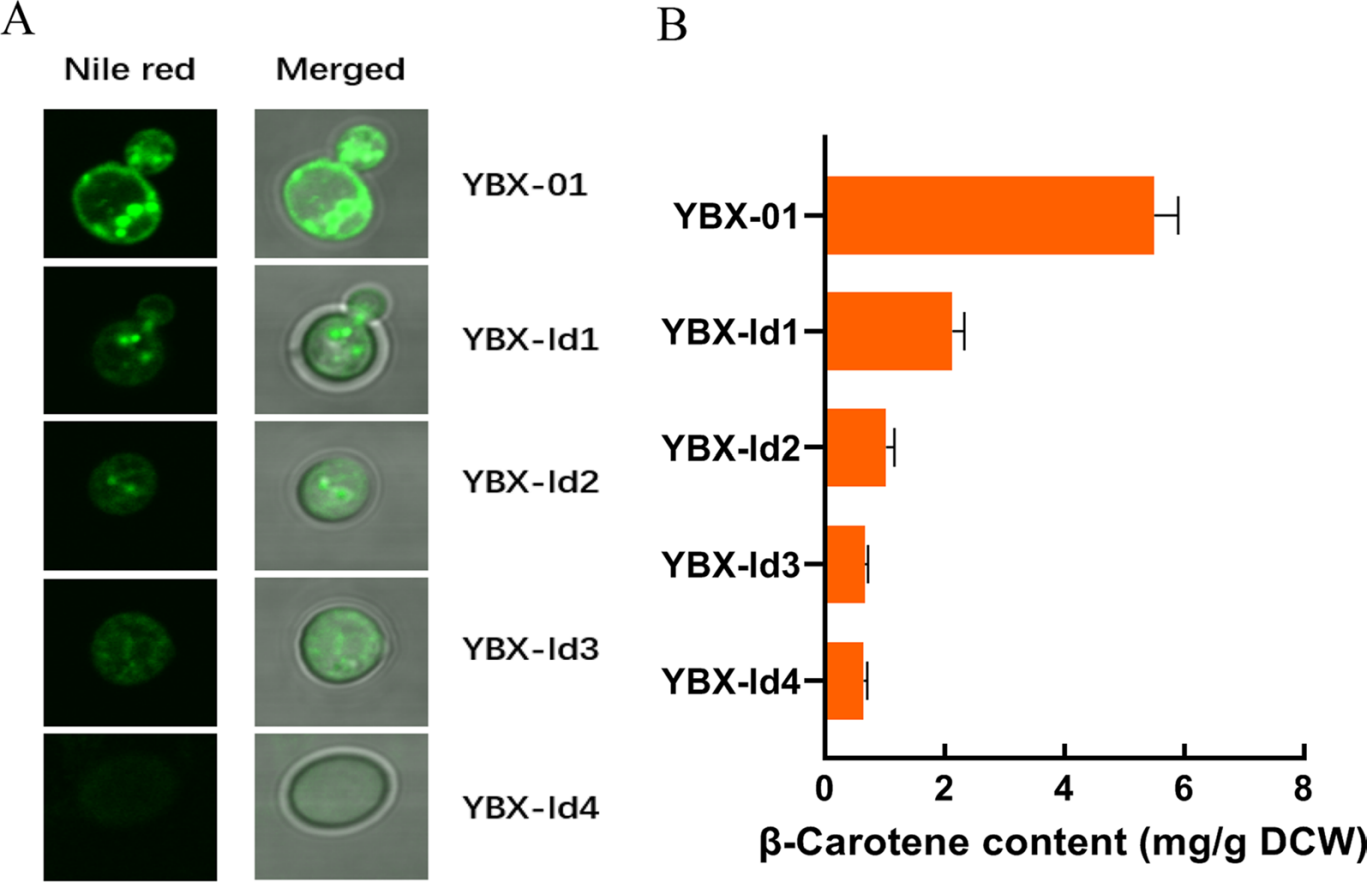
Effect of deleting lipid droplets synthesis genes on intracellular LDs formation observed by laser confocal images (**A**) and β-carotene content (**B**). The genes of *DGA1*, *LRO1*, *ARE1* and *ARE2* were successively disrupted in the parent strain YBX-01 resulting in strains YBX-ld1, ld2, ld3 and ld4, respectively. Data are the means ± standard deviations of triplicate experiments

LDs mainly consist of triacylglycerol (TAG) and sterol esters (SE), in which TAG is located in the core of LDs and is the major lipid species [22]. The content of TAG in strain YBX-01 was found to reach 32.2 mg/g DCW, which is 5.37-fold greater than the wild-type strain YBX-B (Additional file 1: Fig. S2). Correspondingly, three key genes associated with TAG synthesis, including *ACC1* (encoding acetyl-CoA carboxylase), *PAH1* (encoding phosphatidate phosphatase) and *DGA1* (encoding diacylglycerol acyltransferase) were transcriptionally induced, especially after 24 h of the fermentation (Additional file 1: Fig. S2). Next, we successively knocked out four key genes controlling LDs synthesis in strain YBX-01, to investigate the variation of LDs synthesis on β-carotene accumulation. In *S. cerevisiae, DGA1* and *LRO1* (encoding acyltransferase) are responsible for the synthesis of TAG, while *ARE1* and *ARE2* encoding acyl-CoA: sterol acyltransferase contribute to SE synthesis [23]. The deletions generated four strains (YBX-ld1, ld2, ld3 and ld4), respectively. The confocal microscope shows that the number of LDs in engineered strains gradually decreased, and no LDs were observed in the quadruple disruption strain YBX-ld4 (Fig. 2A). As expected, the content of β-carotene correspondingly decreased. There is only 14.8% of β-carotene left in strain YBX-ld4 (Fig. 2B). Noticeably, the strain YBX-ld1 with *DGA1* disruption showed a 61.3% decrease compared to strain YBX-01, confirming that *DGA1* is the major gene controlling TAG synthesis [3]. These results verified the vital role of LDs in storing β-carotene, and proved that LDs are the major storage space of β-carotene in recombinant *S. cerevisiae*, thus LDs were the engineer targets for increasing β-carotene accumulation.

### The limitation of modulating LDs key genes for β-carotene overproduction

**Fig. 3:**
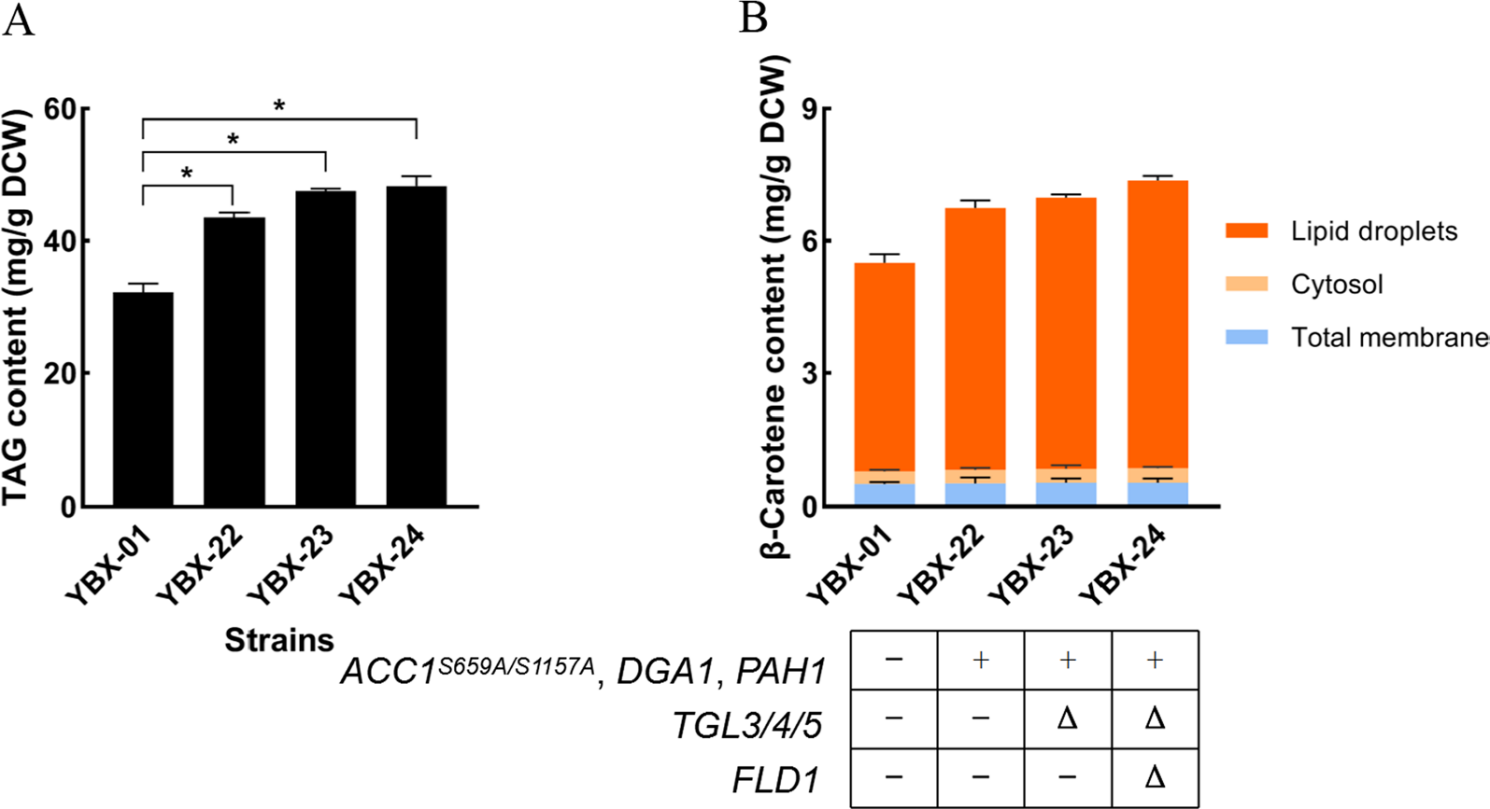
Effect of engineering LDs related genes on intracellular TAG (**A**) and β-carotene content (**B**). YBX-01: the parent strain; YBX-22: the strain with overexpression of *ACC1*, *PAH1* and *DGA1,* derived from strain YBX-01; YBX-23: the strain with disruption of *TGL3*, *TGL4* and *TGL5*, derived from YBX-22; YBX-24: the strain with disruption of *FLD1*, derived from YBX-23. Data are the means ± standard deviations of triplicate experiments. *P < 0.05

Genetic modulating the genes of LDs formation can promote lycopene accumulation [17]. Acetyl-CoA, the starting molecule of TAG biosynthesis, is the central metabolite in fatty acid biosynthesis. *ACC1* is responsible for encoding acetyl-CoA carboxylase to form malonyl-CoA, the first committed and critical step in fatty acid metabolism. The introduction of two site mutations in *ACC1* at Ser659 and Ser1157 results in a three-fold enhancement of *ACC1* activity and increase total fatty acid content [24]. To increase the level of LDs synthesis, we engineered *ACC1* in strain YBX-01 by introducing two site mutations (Ser659Ala and Ser1157Ala) through fusion PCR. The obtained *ACC1*^*S659A/S1157A*^ was linked with the strong constitutive promoter *P*_*TEF1*_*.* Additionally, the original promoters of *PAH1* and *DGA1* were substituted with inducible *P*_*GAL1*_ promoter, and the strain YBX-22 with overexpression of *ACC1*, *PAH1* and *DGA1* was constructed. These modifications led to increased intracellular TAG content from 32.2 to 43.5 mg/g DCW (Fig. 3A), and increased β-carotene content from 5.5 to 6.75 mg/g DCW, which is a 22.7% increase compared to the control strain (Fig. 3B). Through separating LDs, cytoplasm and membrane components, we found that the proportion of β-carotene in LDs increased from 85.5 to 87.6%, while the content in the cytoplasm and membrane system did not change significantly, indicating that almost all of the increased β-carotene was distributed in LDs.

LDs can be cleaved by TAG lipase and SE hydrolase when cells need energy in times of scarcity. To further improve LDs formation, three genes *TGL3*, *TGL4* and *TGL5*, encoding TAG lipase and SE hydrolase [25] were disrupted in strain YBX-22 resulting in strain YBX-23. The content of TAG in strain YBX-23 consequently increased from 43.5 mg/g DCW to 47.5 mg/g DCW (Fig. 3A), and the β-carotene content increased from 6.75 mg/g DCW to 6.98 mg/g DCW (Fig. 3B). A previous study indicated that the disruption of *FLD1* improved lipid levels, LD clustering, and favored the formation of larger LDs [26]. *FLD1* was deleted in strain YBX-23, resulting in strain YBX-24, in which TAG content was further increased to 48.3 mg/g DCW (Fig. 3A). Accordingly, β-carotene content increased to 7.37 mg/g DCW (Fig. 3B). After separation of LDs, we found that the percentage of β-carotene in LDs increased to 88.2%.

Collectively, the above results indicated that engineering the genes associated with LDs synthesis, size and degradation can lead to 50% increase of TAG content, which resulted in 34% increase in β-carotene content compared to the original strain YBX-01. This value was slightly higher than the data reported by Ma et al. that showed a 25% increase of lycopene through engineering fatty acid synthesis and TAG metabolism. Interestingly, when the same strategy was applied to the wild-type strain YBX-B, 180.6 mg/g DCW of TAG was achieved which is 2.74 fold greater than that of strain YBX-24. The discrepancy is possibly due to the competition for precursor (acetyl-CoA) between β-carotene and TAG biosynthesis in strain YBX-24. Obviously, the unilateral improvement of LDs formation is not conducive to β-carotene synthesis, vice versa. Another explanation might be that the promotion of genetic modification on LDs synthesis is limited, considering that LDs is a specific organelle for decreasing the cytotoxicity and will largely be synthesized only when excessive lipophilic products accumulated in cells [23]. Thus, it is essential to design a more effective strategy to address this dilemma.

### The dual regulation of oleic acid for promoting LDs and metabolic pathways

**Fig. 4:**
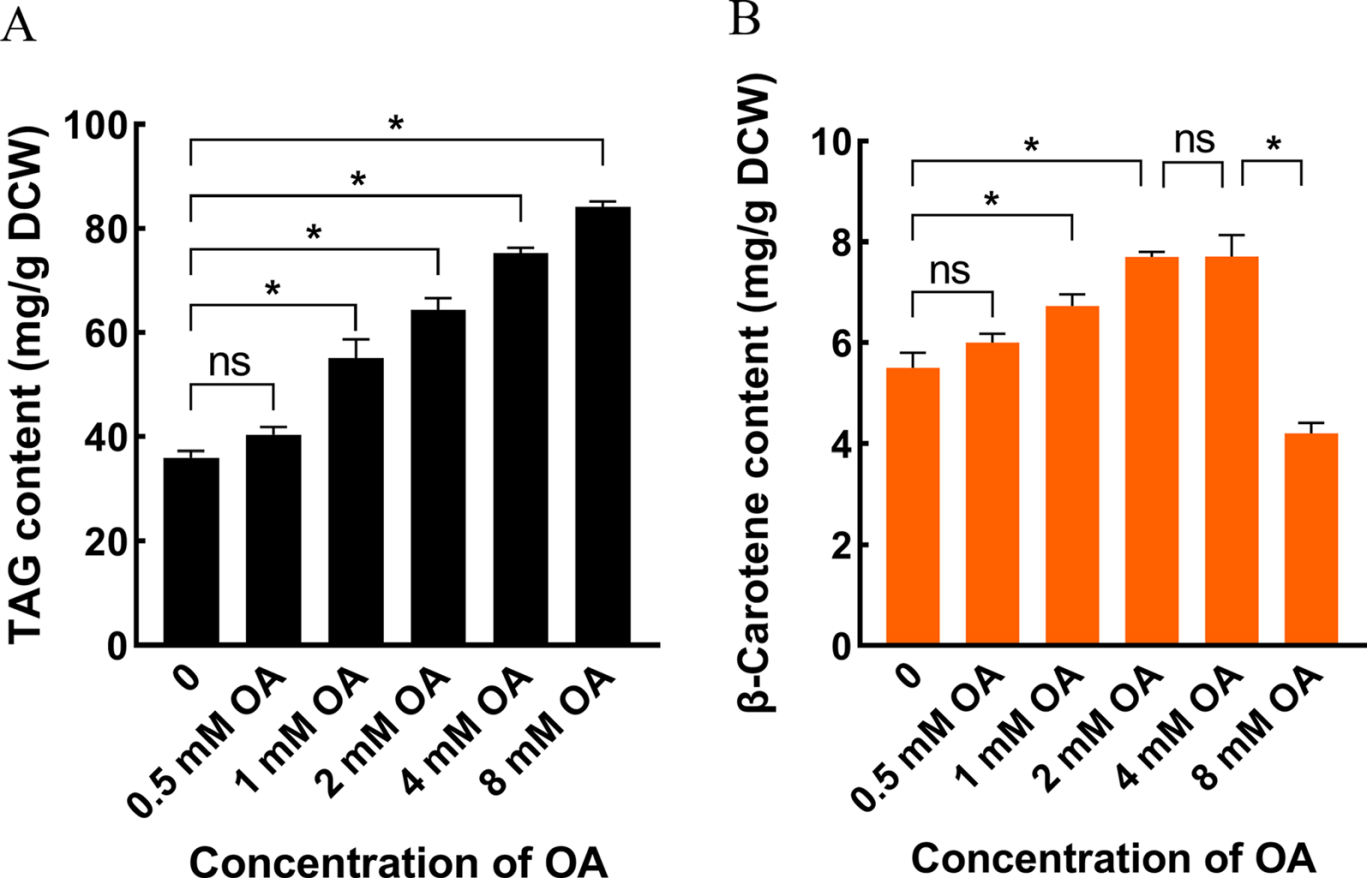
Effects of adding different concentration of oleic acid on intracellular TAG (**A**) and β-carotene content (**B**) in parent strain YBX-01. Data are the means ± standard deviations of triplicate experiments. *P < 0.05

In this work, we focused on OA and aimed to simultaneously promote LDs formation and drive the metabolic flux of MVA to β-carotene pathway, considering that OA has multiple beneficial physiological functions. Firstly, supplementation of OA can dramatically improve the synthesis of LDs in *S. cerevisiae* [23], which additionally save more acetyl-CoA for β-carotene synthesis because cells don’t need de novo synthesis of UFAs. Secondly, the incorporation of UFAs into cell membrane increases the flexibility of cell membrane and relieves carotenoid-induced membrane stress [15, 27]. Thirdly, OA can bind the fatty acid regulatory (FAR) region in the upstream promoter of a specific gene to regulate its expression [28]. Thus, it is possible to mine OA-repressible promoters and substitute *ERG9* original promoter to dynamically down-regulate *ERG9* expression, and resulted in improved β-carotene synthesis by driving the metabolic flux towards the β-carotene biosynthetic pathway. Due to the fact that excessive OA cause lipotoxicity to cells, the optimal concentration of OA on LDs formation should be identified firstly. Thus, 0.5, 1, 2, 4 and 8 mM OA was supplemented into the medium after 12 h of fermentation when strain YBX-01 started the synthesis of β-carotene, respectively. Biomass, TAG and β-carotene content were determined. The results show that the cell growth (Additional file 1: Fig. S3) and TAG metabolism (Fig. 4A) were improved by OA addition. When 2 mM (64.4 mg/g DCW) of OA was used, 78.9% increase of TAG content was achieved compared to the control. This value was 33.3% greater than the engineered strain YBX-24, confirming the high-efficient promotion of exogenous oleic acid on LDs formation. As expected, β-carotene content increased to 7.5 mg/g DCW, which is 36.4% greater than that of the control (Fig. 4B). Additionally, the membrane fluidity of cells was improved by 2.1 fold relative to the control strain through determining the value of fluorescence anisotropy, something that is consistent with our previous work [15]. No significant differences were observed in β-carotene content between 2 and 4 mM OA addition. Noticeably, the addition of 8 mM OA caused a large decrease in β-carotene content (4.2 mg/g DCW), with only 76.4% of the control trial, indicating that lipotoxicity occurred at this concentration. Thus, 2 mM OA was chosen in the following work.

To mine OA-repressible promoters, the transcriptome analysis was adopted to compare mRNA levels in YBX-01 after treated with 2 mM of OA for 2 h and 12 h relative to the control without addition (Additional file 1: Table S4). Five genes (*OLE1*, *MGA2*, *IZH1*, *IZH4* and *YDR274C*) were further selected as potential candidates since their mRNA levels were greatly decreased during the fermentation based on quantitative real-time PCR (qRT-PCR) analysis (Additional file 1: Fig. S4). *OLE1* encodes Delta (9) fatty acid desaturase and is involved in UFAs synthesis, and *MGA2* encoding endoplasmic reticulum membrane protein, participates in the transcriptional regulation of *OLE1* gene as a transcription factor [29]. *IZH1* and *IZH4* encode membrane proteins and associated with zinc ion homeostasis, which are transcriptionally down-regulated when cells are treated with UFAs [15]. *YDR274C* encodes putative protein of unknown function. We cloned their promoters and replaced the original *ERG9* promoter in strain YBX-01, respectively, and generated five strains, including YBX-01-IZH1, YBX-01-IZH4, YBX-01-MGA2, YBX-01-YDR274C and YBX-01-OLE1. The reference strain YBX-01 and the five engineered strains were then examination of cell growth and β-carotene synthesis after 2 mM oleic acid was added.

**Fig. 5:**
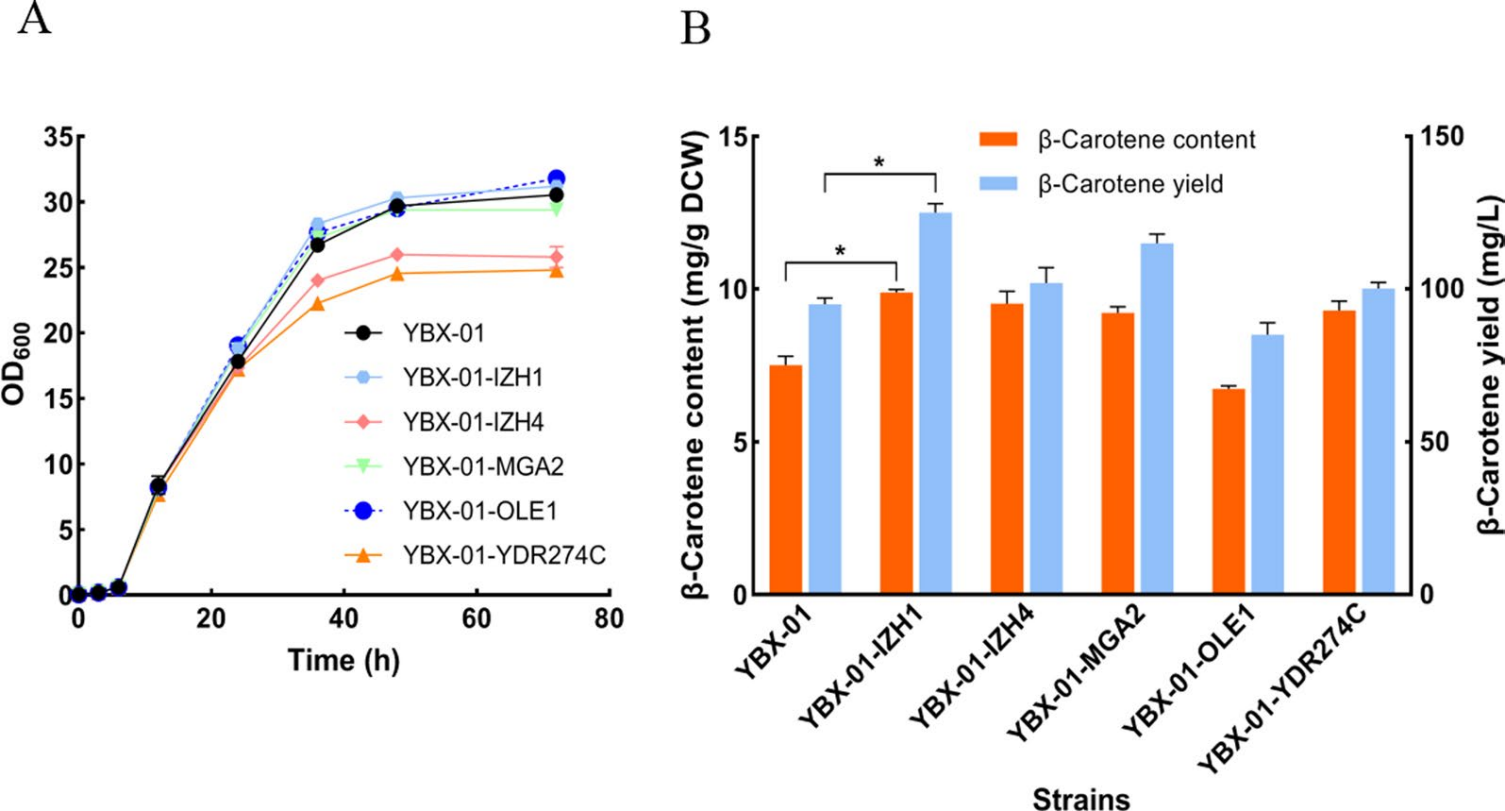
The cell growth (**A**) and β-carotene production (**B**) of different engineered strain with 2 mM oleic acid addition. YBX-01: the parent strain; the original *ERG9* promoter in parent strain YBX-01 was replaced by *IZH1*, *IZH4, MGA2*, *OLE1* and *YDR274C* promoters resulting in respective strains YBX-01-IZH1, YBX-01-IZH4, YBX-01-MGA2, YBX-01-OLE1 and YBX-01-YDR274C. Data are the means ± standard deviations of triplicate experiments. *P < 0.05

As shown in Fig. 5A, except for YBX-01-IZH4 and YBX-01-YDR274C, the resulting strains showed a similar growth pattern to that of the reference strain YBX-01, indicating that the modulation of *ERG9* expression controlled by IZH1, MGA2 and OLE1 promoters dose not interfere with cell growth. Except for strain YBX-01-OLE1, the other four strains showed increase in β-carotene production (Fig. 5B), especially strain YBX-01-IZH1 that exhibited the highest increase, the content and yield increased to 9.88 mg/g DCW and 102 mg/L, which is 31.7% and 32.6% higher than strain YBX-01. The decrease of β-carotene in YBX-01-OLE1 was ascribed to induced transcription of *ERG9*, which led to activation of ergosterol synthesis, as later confirmed. The content of β-carotene in YBX-01-IZH4 and YBX-01-YDR274C was comparable with YBX-01-IZH1, but due to the decreased biomass, the production of β-carotene was only 81.6% and 80.2% of YBX-01-IZH1, respectively.

**Fig. 6:**
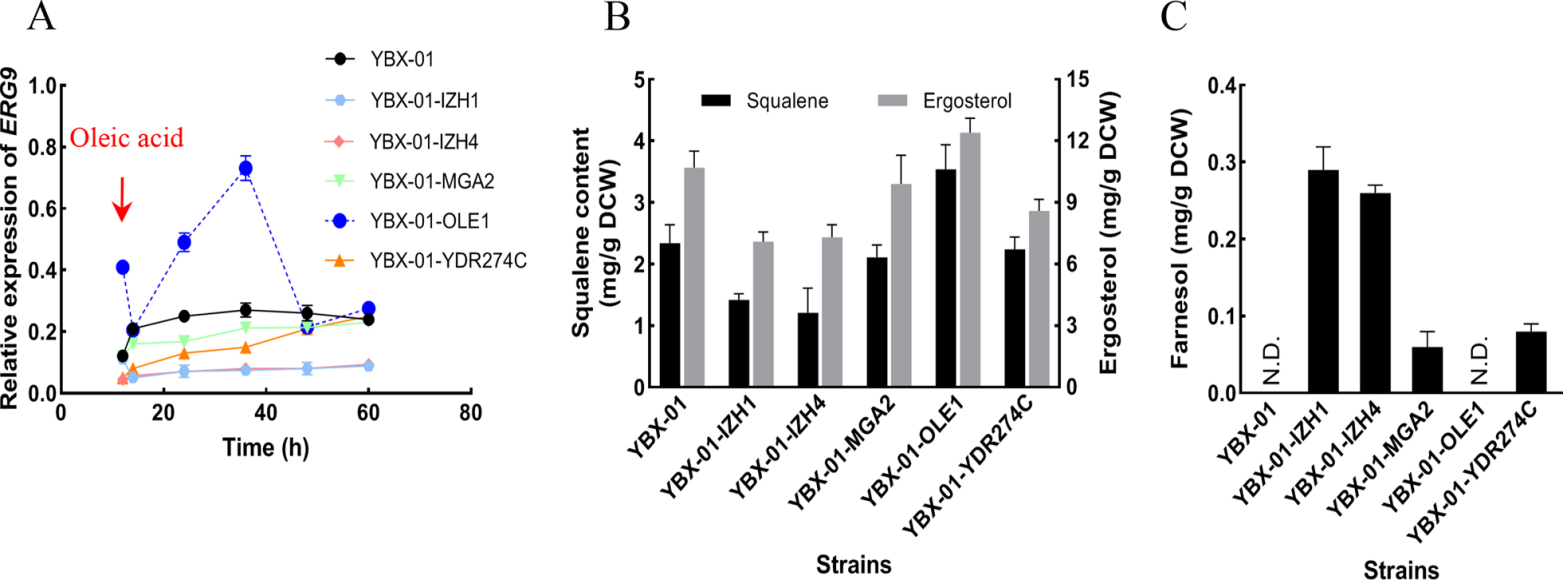
Relative expression of *ERG9* (**A**), squalene, ergosterol (**B**) and farnesol (**C**) contents in different engineered strain with 2 mM oleic acid addition. YBX-01: the parent strain; the original *ERG9* promoter in parent strain YBX-01 was replaced by *IZH1*, *IZH4, MGA2*, *OLE1* and *YDR274C* promoters resulting in respective strains YBX-01-IZH1, YBX-01-IZH4, YBX-01-MGA2, YBX-01-OLE1 and YBX-01-YDR274C. Data are the means ± standard deviations of triplicate experiments

To verify the effect of OA-repressible promoters at molecular level, the expressions of *ERG9* in the strains was determined and compared with the parent strain YBX-01, in which, the expressions remained unchanged after 14 h (Fig. 6A). In strain YBX-01-IZH1 and YBX-01-IZH4, *ERG9* expression was low until the end of the fermentation. *ERG9* mRNA level under the control of *P*_*MGA2*_, although slightly lower, was comparable to that of strain YBX-01. In strain YBX-01-YDR274C, *ERG9* was down-regulated at first, but slowly increased, and no significant differences were observed after 60 h compared to strain YBX-01. Unexpectedly, *ERG9* expression in YBX-01-OLE1 showed a sharp increase after OA addition, and was 2.7 times higher than that of strain YBX-01 at 36 h, after which it decreased to the comparable level of YBX-01 until the end of the fermentation, implying that ergosterol synthesis was activated, which could explain the rise of ergosterol and the descend of β-carotene content. However, the detailed mechanism needs to be further explored.

It is known that the down-regulation of *ERG9* would interfere with the metabolic balance of MVA pathway, and result in the accumulation of intermediate metabolites, such as farnesol, which is a disadvantage for cell growth and product synthesis due to its toxicity [10]. The contents of downstream and intermediate metabolites in different strains was determined, including squalene and ergosterol (Fig. 6B), and farnesol (Fig. 6C). Except for YBX-01-OLE, the contents of squalene and ergosterol in engineered strains were lower than strain YBX-01. Especially in strains YBX-01-IZH1 and YBX-01-IZH4, 39.3% and 48.3%, and 33.6% and 31.8% reduction observed, respectively. This confirmed the efficiency of *P*_*IZH1*_ and *P*_*IZH4*_ on down-regulating *ERG9* expression. Concerning the intermediate metabolite farnesol, similar to previous work results, farnesol was not detected in reference strain YBX-01, while 0.29 and 0.26 mg/g DCW of farnesol were produced in strains YBX-01-IZH1 and YBX-01-IZH4, respectively, indicating that this intermediate metabolite cannot be effectively consumed by inducible biosynthetic pathway of β-carotene. No farnesol accumulated in strain YBX-01-OLE1, corresponding to the highest amount of squalene and ergosterol. These results collectively indicated that *P*_*IZH1*_ is a suitable promoter capable to dynamically down-regulate *ERG9* expression, which can drive the metabolic flux towards carotenoid pathway to improve β-carotene synthesis.

### The introduction of constitutive metabolic pathways to balance the utilization of farnesol

To further improve β-carotene production, it is vital to facilitate further conversion of precursor farnesol to β-carotene. The accumulation of farnesol in strain YBX-01-IZH1 is possibly due to the imbalance in expression time between *ERG9* down-regulation and β-carotene biosynthesis. In this work, in order to reduce the toxicity of carotenoid on cell growth, we introduced an inducible GAL promoter to drive β-carotene biosynthesis [10]. This strategy guarantees that β-carotene will start to synthesize after 12 h when glucose is exhausted, and achieve the separation of the cell growth stage from the product accumulation stage. However, using this strategy, before the synthesis of β-carotene, the engineered strain already synthesized farnesol.

To address this issue, we introduced another β-carotene synthesis pathway driven by glucose and medium-strength promoter *PYK1* and *TPI1*, aimed to initiate β-carotene biosynthesis before 12 h during which glucose is available in the medium. Additionally, to reduce the loss of farnesol by other side pathway, we knocked out *DPP1* and *LPP1* genes in YBX-01-IZH, involved in the hydrolysis of isoprene pyrophosphate [30], resulting in strain YBX-41. The biomass, squalene, farnesol and β-carotene content in YBX-01-IZH1 and YBX-41 were determined after 72 h of cultivation (Table 1). The results show that the biomass of YBX-41 was slightly affected compared to YBX-01-IZH1, and squalene content was further decreased to 1.2 mg/g DCW from 1.42 mg/g DCW, while farnesol was no longer detectable in strain YBX-41. Correspondingly, β-carotene content and yield increased to 11.4 mg/g DCW and 142 mg/L, which is 15.6% and 13.6% greater than those of strain YBX-01-IZH1. This verified that the introduction of extra constitutive synthesis pathway facilitates the complete consumption of farnesol, which resulted in additional increase of β-carotene production with the introduction of inducible synthesis pathway. It is reasonable to believe that by using other metabolic engineering strategies, such as supply of more precursors, energy and reducing equivalents, could further increase the production of β-carotene.

**Table 1:** The biomass, β-carotene and metabolites content in strains YBX-01-IZH1 and YBX-41

Strains	Biomass (OD_600_)	Squalene (mg/g DCW)	Farnesol (mg/g DCW)	β-Carotene content (mg/g DCW)	β-Carotene yield (mg/L)
YBX-IZH1	31.20 ± 0.13	1.41 ± 0.11	0.29 ± 0.03	9.88 ± 0.10	125 ± 2.96
YBX-41	30.30 ± 0.27	1.20 ± 0.08	N.D.	11.42 ± 0.21	142 ± 1.73

N.D. represents not detected

## Conclusion

To improve β-carotene production by engineered *S. cerevisiae*, a strategy that simultaneously enlarges β-carotene storage and enhances the pathway flux to carotenoid synthesis was developed by utilizing exogenous OA combined with metabolic engineering approaches. Quantitative proof that LDs are major storage locations of β-carotene laid the foundation of attempting to improve β-carotene accumulation through promoting LDs formation. *IZH1* promoter is able to dynamically down-regulate *ERG9* expression, and divert the metabolic flux to β-carotene pathway to increase β-carotene accumulation in the presence of OA. Moreover, the membrane stress induced by carotenoid accumulation were largely relieved in this condition. An extra constitutive β-carotene synthesis pathway was induced to facilitate further conversion of precursor farnesol to β-carotene. The final strain could produce 11.4 mg/g DCW and 142 mg/L of β-carotene, which is 107.3% and 49.5% higher than those of the original strain, respectively. This strategy presented here can be applied in the overproduction of other heterogeneous FPP-derived hydrophobic compounds with similar synthesis and storage mechanisms in *S. cerevisiae*.

## Methods and materials

### Strains, media and reagents

*Escherichia coli* DH5α high efficiency competent cells were obtained from Tiangen biotech, Beijing, China for gene cloning and plasmid construction. The wild type strain *S. cerevisiae* FY1679-01B (*MATα*; ura3-52) was used as the host for DNA integration and β-carotene production. LB (Luria–Bertani broth) medium with antibiotics (50 mg/mL of kanamycin) was used for cultivation of recombinant *E. coli*. Yeast extract-peptone-dextrose medium (YPD) was prepared with 1% yeast extract, 2% peptone and 2% dextrose, used for cultivating yeast strains and shake flask fermentation. YPD supplemented with 200 mg/mL geneticin was made for selection of the *KanMX* marker. For selection of yeast strains with excised *KanMX-URA3-PRB322ori* marker, 1 mg/mL 5-fluoroorotic acid SD-FOA (5-FOA) was used to supplement SD complete medium. All restriction enzymes were purchased from Takara (Dalian, China). The standard β-carotene, antibiotics and chemicals were purchased from Sigma (Sigma Aldrich, USA).

### Plasmid and strains construction

All strains and plasmids are listed in Table 2. The primers were ordered from Sangon Biotech (Shanghai, China) and the sequences were provided in Additional file 1: Table S1. DNA fragments, promoters, and homologous arms were PCR amplified from the genomic DNA of *S. cerevisiae* FY1679-01B. Plasmid pUMRI-21 kindly provided by Prof. Hong-wei Yu [31]; it was used as a template for DNA integration into the yeast genome. The detailed protocol for constructing the promoter replacement plasmids can be found in our previous study [12]. To construct β-carotene producing strain and derived engineered strains, pUMRI derivative plasmids were linearized from the junction of homologous arms with corresponding restriction enzymes and integrated into the yeast genome by the lithium acetate/polyethylene glycol/single-stranded carrier DNA transformation method [32]. The recombinant strains were selected by G418 or uracil plates. All transformants were evaluated by genomic DNA PCR using verification primers (Additional file 1: Table S2). After PCR confirmation, the correct colonies were passaged overnight at 30 °C and 220 rpm. Subsequent recombination between the duplicated loxp flanks resulted in 5-FOA resistance due to URA3 excision [33], and 5-FOA-resistant colonies were checked for loss of the targeted marker by replica-plating on YPD and YPD-G418 plates. The promoter replacement schematic in yeast genome can be seen in our previous study [12].

**Table 2:** Plasmid and yeast strains used in this study

Plasmids/strains	Genotype	Source
Plasmids		
pUMRI-21	*loxp-KanMX-URA3-pbr322ori-loxp*, *T*_*ADH1*_*-MCS1-P*_*GAL10*_*-P*_*GAL1*_*-MCS2-T*_*CYC1*_, No Homologous Arms	Lv et al. [31] (KM216411)
pBX-11	*loxp-KanMX-URA3-pbr322ori-loxp*, *ΔDGA1: T*_*ADH1*_*-MCS1-P*_*GAL10*_*-P*_*GAL1*_*-MCS2-T*_*CYC1*_	This study
pBX-12	*loxp-KanMX-URA3-pbr322ori-loxp*, *ΔLRO1: T*_*ADH1*_*-MCS1-P*_*GAL10*_*-P*_*GAL1*_*-MCS2-T*_*CYC1*_	This study
pBX-13	*loxp-KanMX-URA3-pbr322ori-loxp*, *ΔARE1: T*_*ADH1*_*-MCS1-P*_*GAL10*_*-P*_*GAL1*_*-MCS2-T*_*CYC1*_	This study
pBX-14	*loxp-KanMX-URA3-pbr322ori-loxp*, *ΔARE2: T*_*ADH1*_*-MCS1-P*_*GAL10*_*-P*_*GAL1*_*-MCS2-T*_*CYC1*_	This study
pBX-15	*loxp-KanMX-URA3-pbr322ori-loxp*, *ΔTGL3: T*_*ADH1*_*-MCS1-P*_*GAL10*_*-P*_*GAL1*_*-MCS2-T*_*CYC1*_	This study
pBX-16	*loxp-KanMX-URA3-pbr322ori-loxp*, *ΔTGL4: T*_*ADH1*_*-MCS1-P*_*GAL10*_*-P*_*GAL1*_*-MCS2-T*_*CYC1*_	This study
pBX-17	*loxp-KanMX-URA3-pbr322ori-loxp*, *ΔTGL5: T*_*ADH1*_*-MCS1-P*_*GAL10*_*-P*_*GAL1*_*-MCS2-T*_*CYC1*_	This study
pBX-18	*loxp-KanMX-URA3-pbr322ori-loxp*, *ΔFLD1: T*_*ADH1*_*-MCS1-P*_*GAL10*_*-P*_*GAL1*_*-MCS2-T*_*CYC1*_	This study
pBX-19	*loxp-KanMX-URA3-pbr322ori-loxp*, *ΔHO: T*_*ADH1*_*-MCS1-P*_*GAL10*_*-P*_*GAL1*_*-MCS2-T*_*CYC1*_	This study
pBX-20	*loxp-KanMX-URA3-pbr322ori-loxp*, *ΔHO: T*_*ADH1*_*-PAH1-P*_*GAL10*_*-P*_*GAL1*_*-DGA1-T*_*CYC1*_	This study
pBX-21	*loxp-KanMX-URA3-pbr322ori-loxp*, *P*_*TEF1*_*-ACC1 *^*S659A*, *S1157A*^* -T*_*CYC1*_	This study
pBX-22	*loxp-KanMX-URA3-pbr322ori-loxp*, *ΔTy4: P*_*TEF1*_*-ACC1 *^*S659A*, *S1157A*^* -T*_*CYC1*_	This study
pBX-OLE1pro	*ΔP*_*ERG9*_*::P*_*OLE1*_	This study
pBX-MGA2pro	*ΔP*_*ERG9*_*::P*_*MGA2*_	This study
pBX-IZH1pro	*ΔP*_*ERG9*_*::P*_*IZH1*_	This study
pBX-IZH4pro	*ΔP*_*ERG9*_*::P*_*IZH4*_	This study
pBX-YDR274Cpro	*ΔP*_*ERG9*_*::P*_*YDR274C*_	This study
pBX-23	*loxp-KanMX-URA3-pbr322ori-loxp, T*_*ADH1*_*-MCS1-P*_*PYK1*_*-P*_*TPI1*_*-MCS2-T*_*CYC1*_	This study
pBX-24	*Δdpp1::T*_*ADH1*_*-CrtYB-P*_*PYK1*_*-P*_*TPI1*_*-CrtI-T*_*CYC1*_*, loxp-KanMX-URA3-pbr322ori-loxp*	This study
pBX-25	*Δlpp1::T*_*ADH1*_*-MCS-P*_*PYK1*_*-P*_*TPI1*_*-CrtE-T*_*CYC1*_*, loxp-KanMX-URA3-pbr322ori-loxp*	This study
Strains		
YBX-ld1	YBX-01,*Δdga1*	This study
YBX-ld2	YBX-ld1,*Δlro1*	This study
YBX-ld3	YBX-ld2, *Δare1*	This study
YBX-ld4	YBX-ld3, *Δare2*	This study
YBX-22	YBX-01,*Δho::T*_*ADH1*_*-DGA1-P*_*GAL10*_*-P*_*GAL1*_*-PAH1-T*_*CYC1*_; *ΔTy4::P*_*TEF1*_*-ACC1*^*S659A*, *S1157A*^*-T*_*CYC1*_	This study
YBX-23	YBX-22, *Δtgl3*, *Δtgl4*, *Δtgl5*	This study
YBX-24	YBX-23, *Δfld1*	This study
YBX-01-OLE1	YBX-01, *ΔP*_*ERG9*_*::P*_*OLE1*_	This study
YBX-01-MGA2	YBX-01, *ΔP*_*ERG9*_*::P*_*MGA2*_	This study
YBX-01-IZH1	YBX-01, *ΔP*_*ERG9*_*::P*_*IZH1*_	This study
YBX-01-IZH4	YBX-01, *ΔP*_*ERG9*_*::P*_*IZH4*_	This study
YBX-01-YDR274C	YBX-01, *ΔP*_*ERG9*_*::P*_*YDR274C*_	This study
YBX-41	YBX-01-IZH1, *Δdpp1::T*_*ADH1*_*-CrtYB-P*_*PYK1*_*-P*_*TPI1*_*-CrtI-T*_*CYC1*_, *Δlpp1::P*_*TPI1*_*-CrtE-T*_*CYC1*_	This study

The description of the genotype in the table is based on the parent strain YBX-01

### Cultivation conditions

For β-carotene shake-flask fermentation, the recombinant yeast colonies were inoculated into 5 mL YPD medium and cultured at 30 °C on a rotary shaker (220 rpm) overnight. The seed cultures were inoculated into 250 mL flasks containing 50 mL YPD medium at an initial OD_600_ of 0.05 and cultured under the same conditions for 72 h. Cell growth was determined by the absorbance of OD_600_ and dry cell weight (DCW). Cells were harvested after 72 h of cultivation to determine β-carotene production and other metabolites.

### Quantification of β-carotene, TAG, farnesol, ergosterol and squalene

The intracellular β-carotene was extracted using hot HCl-acetone [10]. The analyses of β-carotene were performed on a HPLC system (Agilent 1200 LC) equipped with a C18 column (4.6 mm × 150 mm) and the UV/VIS signals were detected at 450 nm. The mobile phase consisted of acetonitrile-methanol-isopropanol (50:30:20 v/v) with a flow rate of 1 mL/min at 40 °C. TAG content in yeast was determined using assay kit (Order NO. D799795), purchased from Sangon Biotech (Shanghai, China). The quantification of farnesol in *S. cerevisiae* was based on the method of Song [34] with a slight modification. In brief, methanol, hexane, and fermentation broth (1:1:1 v/v/v) were mixed with a vortex mixer for 1.5 min and centrifuged. The top hexane layer was transferred into a 1.5 mL centrifuge tube and then use nitrogen to blow n-hexane completely volatilize. 500 μL of methanol was then added to dissolve farnesol, and filter with 0.22 μm syringe filter and analyzed by HPLC equipped with C18 column (5 μm, 4.6 mm × 150 mm). The mobile phase was: 90% methanol aqueous solution; flow rate: 1 mL/min, column temperature: 40 °C, the injection volume: 20 μL, and the UV detector detects at 206 nm. Ergosterol content was measured as described by Sun et al. [35]. Squalene was quantified using HPLC according to the procedure detailed in Xie et al. [10]. The contents of farnesol, ergosterol and squalene were expressed as the mg/g dry cell weight. All measurements were performed in triplicate.

### Separation and purification of LDs

The separation and purification protocol of LDs in *S. cerevisiae* was according to the method reported by Ding et al. [21]. In brief, cells representing 300 mg of DCW were re-suspended in buffer A (pH 7.8) containing 20 mM tricine and 250 mM sucrose and homogenized in a French press at 1,500 bar. The lysate was centrifuged for 10 min at 3.000×*g* to remove cell debris. The supernatant was overlaid with buffer B (pH 7.4) containing 20 mM HEPES, 100 mM KCl and 2 mM MgCl_2_ and centrifuged for 1 h at 100,000×*g* in a Beckman Coulter Optima LE80K ultracentrifuge. The floating lipid body fraction was separated, and each phase floating phase (LDs), interphase (cytosol), pellet (total membrane phase) was extracted three times with extraction solvent. Adjusted the extracted β-carotene to the required volume by nitrogen blowing, and quantified β-carotene by liquid chromatography.

### Observation of LDs by electron and confocal microscopy

For observation of lipid droplets by transmission electron microscopy, the sample of yeast was prepared according to previous literature [36]. For confocal microscopy, the lipid droplets staining and image acquisition were based on the method of Lv et al. [37] with a slight modification. Yeast cells were harvested at 5.0 (OD_600_), then washed and re-suspended with 500 μL PBS, and 520 μL Nile red staining solution (0.1% Nile red in DMSO) was added. The solution was thoroughly mixed and incubated in dark at room temperature for 10 min. Images were acquired with an Olympus AX70 Fluorescence Microscope (Olympus, Tokyo, Japan). Fluorescence excitation was at 488 nm.

### Transcriptome and qRT-PCR analysis

Total RNA was isolated from the harvested yeast cells by using the HiPure Yeast RNA Kit (Magen, Guangzhou, China) as recommended by the manufacture’s protocol. Residual genomic DNA contamination was removed by an RNase-Free DNase I treatment after RNA purification. The treated total RNA was reversely transcribed using HiScript^®^ II Q RT SuperMix for Qpcr (+gDNA wiper) (Vazyme, Nanjing, China). The purified RNA samples, were determined using an Agilent 2100 Bioanalyzer (Agilent Technologies). The cDNA library construction and sequencing were performed by Novogene Co. Ltd, Beijing, China (http://www.novogene.cn/) using the Illumina HiSeq™ 4000 platform. The clean reads were aligned to the reference genome (http://www.yeastgenome.org/). The RPKM (Fragments per kilobase per million fragments mapped) of each gene was calculated and used to represent the expression level of gene. The primers used in qRT-PCR are shown in Additional file 1: Table S3. The housekeeping gene *ACT1* was used as the reference gene to normalize the different samples. The relative gene expression analysis was performed using the 2^−*ΔΔCT*^ method [38].

## Supplementary Information

**Additional file 1: Figure S1. **Observation of yeast lipid droplets by transmission electron microscopy. **Figure S2.** The intracellular TAG content (A) and the relative expression of lipid droplets synthesis genes (B) between the wild-type strain YBX-B and β-carotene synthesizing strain YBX-01. **Figure S3.** Effects of adding different concentration of oleic acid on cell growth of the parent strain YBX-01. **Figure S4.** The relative expression of target genes in strain YBX-01 after treated with 2 mM of OA for 2 h and 12 h relative to the control without addition by qRT-PCR. **Table S1.** Primers used for plasmid construction in this work. **Table S2.** Primers for genotype identification of promoter replaced strains. **Table S3.** Primers for quantitative real-time PCR in this work. **Table S4.** Down-regulated genes in strain YBX-01 after treated with oleic acid for 2 h and 12 h by transcriptome analysis.
