# Supplementary material for: Dual regulation of lipid droplet-triacylglycerol metabolism and ERG9 expression for improved β-carotene production in Saccharomyces cerevisiae
Source: Microb Cell Fact. 2022 Jan 4;21:3. doi: 10.1186/s12934-021-01723-y (PMC8725481; doi:10.1186/s12934-021-01723-y)
Supplement: Supplementary file 1 — Additional file 1: Figure S1. Observation of yeast lipid droplets by transmission electron microscopy. Figure S2. The intracellular TAG content (A) and the relative expression of lipid droplets synthesis genes (B) between the wild-type strain YBX-B and β-carotene synthesizing strain YBX-01. Figure S3. Effects of adding different concentration of oleic acid on cell growth of the parent strain YBX-01. Figure S4. The relative expression of target genes in strain YBX-01 after treated with 2 mM of OA for 2 h and 12 h relative to the control without addition by qRT-PCR. Table S1. Primers used for plasmid construction in this work. Table S2. Primers for genotype identification of promoter replaced strains. Table S3. Primers for quantitative real-time PCR in this work. Table S4. Down-regulated genes in strain YBX-01 after treated with oleic acid for 2 h and 12 h by transcriptome analysis. [file 12934_2021_1723_MOESM1_ESM.docx]

Additional file

Fig. S1


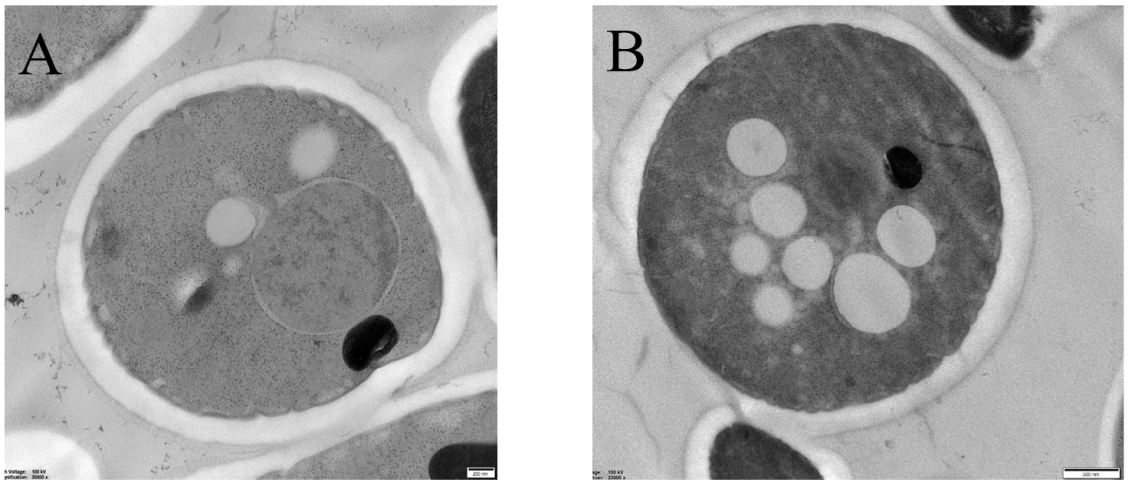


Fig. S1. Observation of yeast lipid droplets by transmission electron microscopy

(A) Transmission electron micrograph of the wild-type strain YBX-B, bar: 200 nm; (B) Transmission electron micrograph of β-carotene synthesizing strain YBX-01, bar: 500 nm

Fig. S2


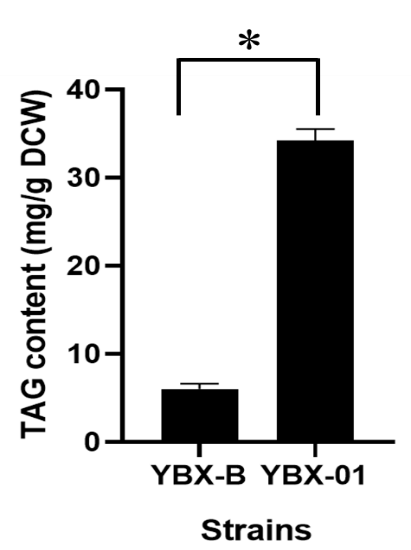


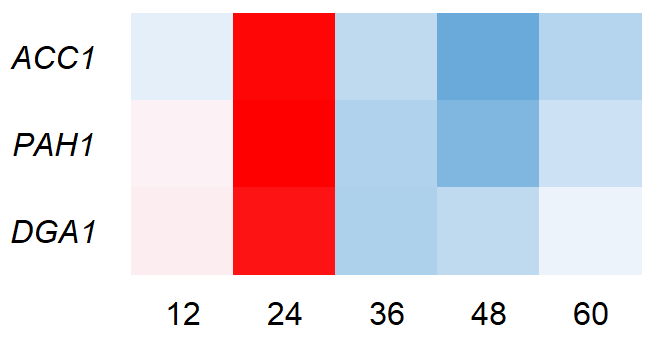

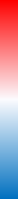


2

1

0


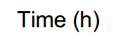


Fig. S2. The intracellular TAG content (A) and the relative expression of lipid droplets synthesis genes (B) between the wild-type strain YBX-B and β-carotene synthesizing strain YBX-01

Fig. S3


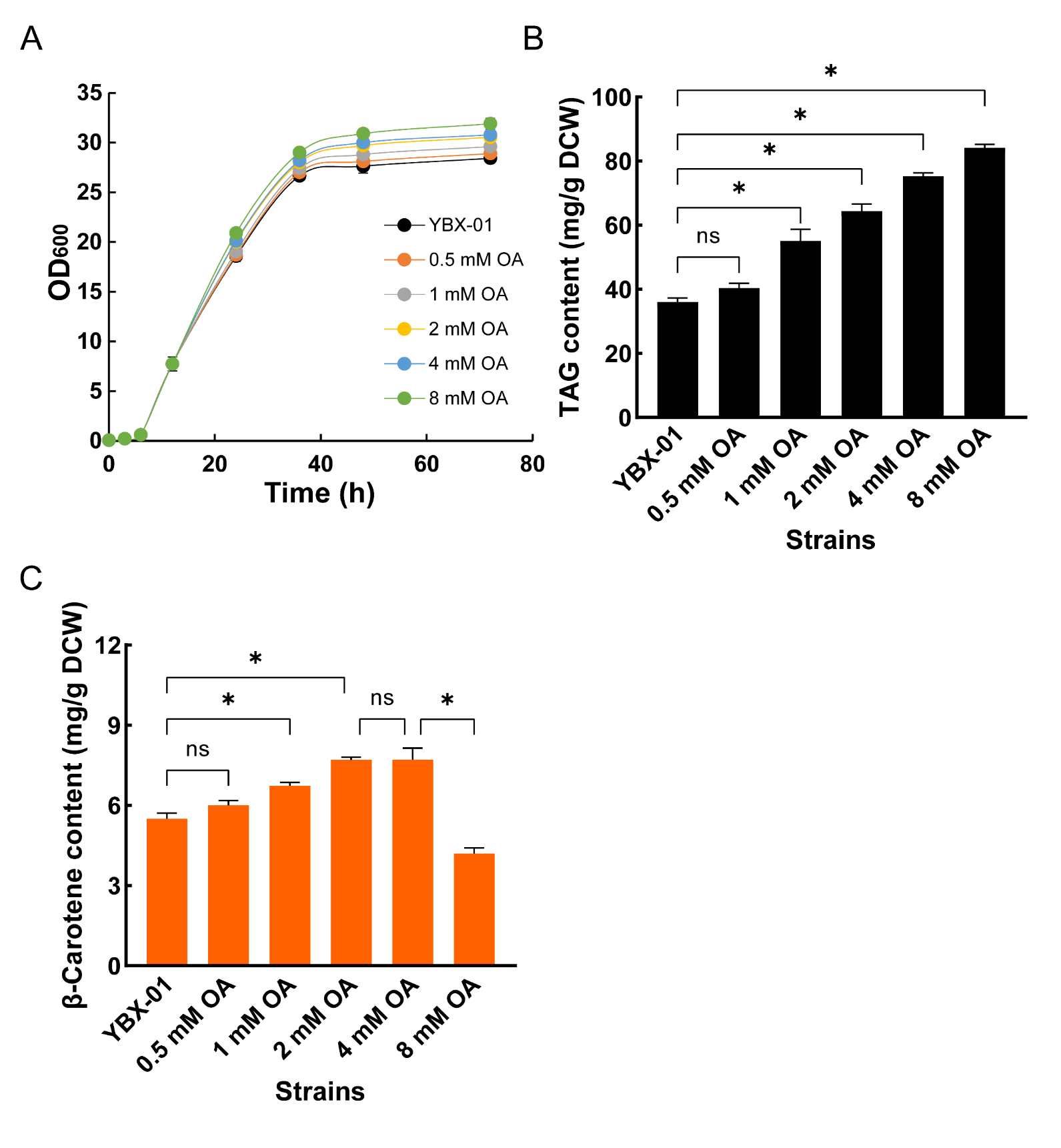


Fig. S3. Effects of adding different concentration of oleic acid on cell growth of the parent strain YBX-01

Fig. S4


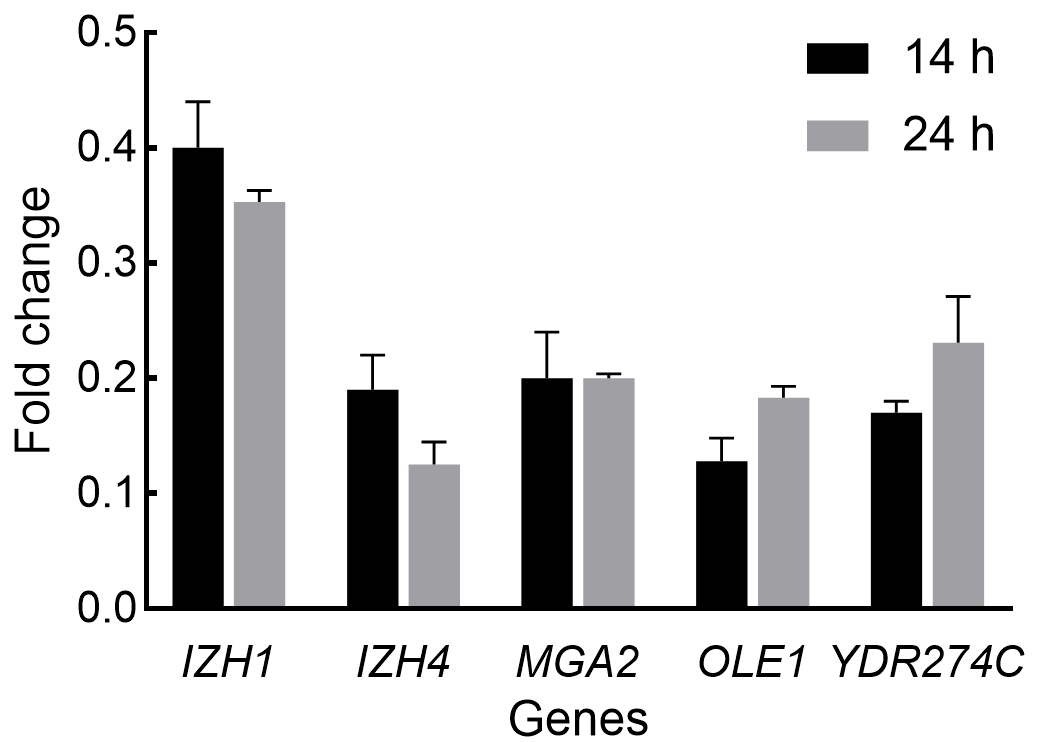


Fig. S4. The relative expression of target genes in strain YBX-01 after treated with 2 mM of OA for 2 h and 12 h relative to the control without addition by qRT-PCR

Table S1 Primers used for plasmid construction in this work

| Primers | Sequence（5’-3’） | Note |
| --- | --- | --- |
| Amplification of structural genes | | |
| CrtYB F (SacI) | GGATGACGACGATAAGATCTGAGCTATGACGGCTCTCGCATATTA | *CrtYB* |
| CrtYB R (SacI) | GGCGAAGAATTGTTAATTAAGAGCTTTACTGCCCTTCCCATCCGC |  |
| CrtI F (XhoI) | AGTTGATTTCCGAAGAAGACCTCGAATGGGAAAAGAACAAGATCAGG | *CrtI* |
| CrtI R (XhoI) | TAGCCGCGGTACCAAGCTTACTCGATCAGAAAGCAAGAACACCAAC |  |
| CrtE F (EcoRI) | GTAAGAATTTTTGAAAATTCGAATTATGGATTACGCGAACATCCT | *CrtE* |
| CrtE R (EcoRI) | GCCGCCCTTTAGTGAGGGTTGAATTTCACAGAGGGATATCGGCTAG |  |
| DGA1 F | GTAAGAATTTTTGAAAATTCGAATTATGTCAGGAACATTCAATGATATAAG | *DGA1* ORF |
| DGA1 R | GCCGCCCTTTAGTGAGGGTTGAATTTTACCCAACTATCTTCAATTCTGCAT |  |
| PAH1 F | CGTCAAGGAGAAAAAACCCCGGATCATGCAGTACGTAGGCAGAG | *PAH1* ORF |
| PAH1 R | CCTATAGTGAGTCGTATTACGGATCTTAATCTTCGAATTCATCTTCGTCGAA |  |
| ACC1 F1 | CGTCAAGGAGAAAAAACCCCGGATCAGTTTCTACCATGAGCGAAGAAAGC | *ACC1* (the first part) |
| ACC1 R1 | AAAAGACCACCATCAGCTAGTTGACGCAGTA |  |
| ACC1 F2 | TACTGCGTCAACTAGCTGATGGTGGTCTTTT | *ACC1* (the second part) |
| ACC1 R2 | CATATGACAAATCTGAAACAGCAACAGCCCTGTTCATA |  |
| ACC1 F3 | TATGAACAGGGCTGTTGCTGTTTCAGATTTGTCATATG | *ACC1* (the third part) |
| ACC1 R3 | CCTATAGTGAGTCGTATTACGGATCCTTTACAAGGTTTATTTCAAAGTCTTCAACAAT |  |
| Amplification of promoters | | |
| TEF1 F | GCCGCCCTTTAGTGAGGGTTGAATTCACACACCATAGCTTCAAAATG | *TEF1* promoter |
| TEF1 R | CTATAGTGAGTCGTATTACGGATCTTGTAATTAAAACTTAGATTAGATTGCTATGCT |  |
| PYK1 pro F | TGACCATACGTAGGAAAGTTTTTCCGGCAAGCTAA | *PYK1* promoter |
| PYK1 pro R | CCTTTAGTGAGGGTTGAATTTGTGATGATGTTTTATTTGTTTTGA |  |
| TPI1 pro F | CGGAAAAACTTTCCTACGTATGGTCATTTCTTC | *TPI1* promoter |
| TPI1 pro R | CCTATAGTGAGTCGTATTACGGATCTTTTAGTTTATGTATGTGTTTTTTGTA |  |
| Amplification of homology arms | | |
| DGA1 left F | GTTGTTGTTGGAAGGCCTATGGCCATATAGGCCTCTCTGCAGGCAACTAAGTT | *DGA1* homologous arm |
| DGA1 left R | AGGGAACAAAAGCTGGAGCTGGCCTTGTAGACTTATTCTCATGCCTTTCG |  |
| DGA1 right F | GGCGTAATAGCGAAGAGGCCTACAAACTACTTATTGGCGTTAGGT |  |
| DGA1 right R | GGCGTAATAGCGAAGAGGCCTACAAACTACTTATTGGCGTTAGGT |  |
| LRO1 left F | ATCAACAAACTGGCAATTGCGGCCATATAGGCCCTCCTCAGGAGTTATTTGACCT | *LRO1* homologous arm |
| LRO1 left R | AGGGAACAAAAGCTGGAGCTGGCCGAATGTGGTTTCTGCTCTCTC |  |
| LRO1 right F | GGCGTAATAGCGAAGAGGCCTTGAGCCAGTGGGTTTCTCA |  |
| LRO1 right R | AGGTCAAATAACTCCTGAGGAGGGCCTATATGGCCGCAATTGCCAGTTTGTTGAT |  |
| ARE1 left F | GCAAAGAGCTCCTAGGTCTATGGCCATATAGGCCCACTATGTATGTGCTGCTCAT | *ARE1* homologous arm |
| ARE1 left R | AGGGAACAAAAGCTGGAGCTGGTCAGTCGACTCCACTCCTTC |  |
| ARE1 right F | GGCGTAATAGCGAAGAGGCCAGATATTTCTACGGCGACTG |  |
| ARE1 right R | ATGAGCAGCACATACATAGTGGGCCTATATGGCCATAGACCTAGGAGCTCTTTGC |  |
| ARE2 left F | GCAACTGTTCTGTGGAGCTAGGCCATATAGGCCGAGACTCGAGAAAGAGGGAAG | *ARE2* homologous arm |
| ARE2 left R | AGGGAACAAAAGCTGGAGCTGGCCCGGAGAAATTGTTCGTTCTCC |  |
| ARE2 right F | GGCGTAATAGCGAAGAGGCCTGAACTGTGTGGCTGAATTGAC |  |
| ARE2 right R | CTTCCCTCTTTCTCGAGTCTCGGCCTATATGGCCTAGCTCCACAGAACAGTTGCA |  |
| TGL3 left F | GAGGAATGAACAGAAGCATCGGGCCATATAGGCCCGCTCCCTTGTTTAATAGCTT | *TGL3* homologous arm |
| TGL3 left R | AGGGAACAAAAGCTGGAGCTGGCCATACACTACACGCAGTATCCA |  |
| TGL3 right F | GGCGTAATAGCGAAGAGGCCAGATTGCTGTGGTACCAAG |  |
| TGL3 right R | AAGCTATTAAACAAGGGAGCGGGCCTATATGGCCCGATGCTTCTGTTCATTCCTC |  |
| TGL4 left F | GGGTTGGTACTAATGCTGGAGGCCATATAGGCCGCGCTTCAAAAAGTCATTAGG | *TGL4* homologous arm |
| TGL4 left R | AGGGAACAAAAGCTGGAGCTGGCTCCACCCTTTCAACATCTGA |  |
| TGL4 right F | GGCGTAATAGCGAAGAGGCCTTCAGGCGTCATAGCATTGATGG |  |
| TGL4 right R | CCTAATGACTTTTTGAAGCGCGGCCTATATGGCCTCCAGCATTAGTACCAACCC |  |
| TGL5 left F | CCTAATGACTTTTTGAAGCGCGGCCTATATGGCCTCCAGCATTAGTACCAACCC | *TGL5* homologous arm |
| TGL5 left R | AGGGAACAAAAGCTGGAGCTGGCCATTTGACTGATGGATCAGCG |  |
| TGL5 right F | CAGGAGTGTTGGAAAGTTCGGGCCTATATGGCCCAGCTCCTTGAATGATAGTACG |  |
| TGL5 right R | CAGGAGTGTTGGAAAGTTCGGGCCTATATGGCCCAGCTCCTTGAATGATAGTACG |  |
| FLD1 left F | AAATAATGAGGACACCCCGCGGCCACTAGGGCCGACTGGAATTACAGCGCAAC | *FLD1* homologous arm |
| FLD1 left R | AGGGAACAAAAGCTGGAGCTGGCCCTGTAATGGACGGGATACATTGA |  |
| FLD1 right F | GGCGTAATAGCGAAGAGGCCTATCACAGGTTGCACTGCATTC |  |
| FLD1 right R | GTTGCGCTGTAATTCCAGTCGGCCCTAGTGGCCGCGGGGTGTCCTCATTATTT |  |
| HO left F | AAATTCAGCGGTCATCACTGGACGTCGACTTAAAATGGCGTGGCAG | *HO* homologous arm |
| HO left R | AGGGAACAAAAGCTGGAGCTGGCCGATGTATCTCATCGCAGGCA |  |
| HO right F | GGCCTCTTCGCTATTACGCCCCATTATGGACGGAATTGTC |  |
| HO right R | CTGCCACGCCATTTTAAGTCGACGTCCAGTGATGACCGCTGAATTT |  |
| Ty4 left F | GTGTATGCAAAATGGACAAGAACGGCCATATAGGCCGCTTTCGCAAATACACCAATG | *Ty4* homologous arm |
| Ty4 left R | AGGGAACAAAAGCTGGAGCTGGACAGCGTTACCAATATGGTATG |  |
| Ty4 right F | GGCGTAATAGCGAAGAGGCGGATAGTGGAACACATTCCAAG |  |
| Ty4 right R | CATTGGTGTATTTGCGAAAGCGGCCTATATGGCCGTTCTTGTCCATTTTGCATACAC |  |
| DPP1 left F | GGAAGCAGTTGAGTAGCTGAGGCCATATAGGCCCCAACGTTGGATAACCTCA | *DPP1* homologous arm |
| DPP1 left R | AGGGAACAAAAGCTGGAGCTGGGTTCGAACGGTTGTTGGTAA |  |
| DPP1 right F | GGCGTAATAGCGAAGAGGCCTTCCCACCCATTGATGATC |  |
| DPP1 right R | TGAGGTTATCCAACGTTGGGGCCTATATGGCCTCAGCTACTCAACTGCTTCC |  |
| LPP1 left F | CACGCCAGATATTAACCGAAGGGCCATATAGGCCCGTGAAACCTGACAACTTATAG | *LPP1* homologous arm |
| LPP1 left R | AGGGAACAAAAGCTGGAGCTGGCTTGTCAAACATGCAGGTCC |  |
| LPP1 right F | GGCGTAATAGCGAAGAGGCATGTTGTCTCTGGAGCTGTT |  |
| LPP1 right R | CTATAAGTTGTCAGGTTTCACGGGCCTATATGGCCCTTCGGTTAATATCTGGCGTG |  |
| The promoter primers of replace *ERG9* original promoter | | |
| OLE1 pro F | TAGGCCTCTTCGCTATTACGCCAGTGATGTTCTGAGGTATTCGTATCGC | *OLE1* promoter |
| OLE1 pro R | AATTGTAATAGCTTTCCCATCTTTGTTGTAATGTTTTAGTGCTGTTT |  |
| MGA2 pro F | TAGGCCTCTTCGCTATTACGCCAGGCCCTCAACGTAGACAAGTC | *MGA2* promoter |
| MGA2 pro R | AATTGTAATAGCTTTCCCATAACGAAATGTTCTGTTCGCC |  |
| IZH1 pro F | TAGGCCTCTTCGCTATTACGCCAGGCAATATCTCAAAGCCATTAAT | *IZH1* promoter |
| IZH1 pro R | GCCAATTGTAATAGCTTTCCCATTTTTTTTCTTCTTTTATCTTGC |  |
| IZH4 pro F | TAGGCCTCTTCGCTATTACGCCAGCGCTTCCATTTGGGGTTATT | *IZH4* promoter |
| IZH4 pro R | AATTGTAATAGCTTTCCCATACTGTCAATATATATGTACCTTTATACC |  |
| YDR274C pro F | TAGGCCTCTTCGCTATTACGCCAGCGACTCGTGAATGATTTTTCGA | *YDR274C* promoter |
| YDR274C pro R | AATTGTAATAGCTTTCCCATGTACGAGGGAAGAGAAGG |  |
| ERG9-FF | CACGACTACGACGTGTACTGGGATCCGGTCTGCAGGGGAGAACTTA | ERG9 F |
| ERG9-FR | GTTATATTAAGGGTTGTCGAGCGCGCAAAACCGATAACGCCTTCC |  |
| ERG9-OLE1-RF | ACTAAAACATTACAACAAAGATGGGAAAGCTATTACAATTGGC | ERG9 R-OLE1 |
| ERG9-MGA2-RF | GGCGAACAGAACATTTCGTTATGGGAAAGCTATTACAATTGGC | ERG9 R-MGA2 |
| ERG9-IZH1-RF | CAAGCAAGATAAAAGAAGAAAAAAAATGGGAAAGCTATTACAATTGGC | ERG9 R-IZH1 |
| ERG9-IZH4-RF | GGTATAAAGGTACATATATATTGACAGTATGGGAAAGCTATTACAATTGGC | ERG9 R-IZH4 |
| ERG9-YDR274C-RF | CCTTCTCTTCCCTCGTACATGGGAAAGCTATTACAATTGGC | ERG9 R-YDR274C |
| ERG9-RR* | TAAGTTCTCCCCTGCAGACCGGATCCCAGTACACGTCGTAGTCGTG |  |

* ERG9-RR: A universal primer for the amplification of ERG9 R from yeast genomic DNA，including fragment ERG9 R-OLE1，ERG9 R-MGA2，ERG9 R-IZH1，ERG9 R-IZH4 and ERG9 R-YDR274.

Table S2 Primers for genotype identification of promoter replaced strains

| Primer | Sequence（5’-3’） | Theoretical band size |
| --- | --- | --- |
| YZJYZ OLE1pro F | TGGGGATGGCACACAAAGGT | around 1050 bp |
| YZJYZ MGA2pro F | ATCTGGCACTTTGTCTCAGGG | around 1140 bp |
| YZJYZ IZH1pro F | CGACCGATGGATGCAGCAAT | around 1100 bp |
| YZJYZ IZH4pro F | CGGACAATTCGGACAGCACT | around 1060 bp |
| YZJYZ YDR274pro F | GGATGCAGATGCCTGGCAAG | around 1070 bp |
| YZJYZ ERG9 R* | CAGTCCAACCCCAGTTGTTCG | -- |

*YZJYZ ERG9 R: a reverse universal primer for verifying the genome of *ERG9* promoter replacement strain，including the strains YBX-01-OLE1, YBX-01-MGA2, YBX-01-IZH1, YBX-01-IZH4 and YBX-01-YDR274

Table S3 Primers for quantitative real-time PCR in this work

| Primer | Sequence（5’-3’） | Product size |
| --- | --- | --- |
| qACT1-F | TGCAAAAGGAAATCACCGC | 98 bp |
| qACT1-R | GATAGAACCACCAATCCAGACG |  |
| qACC1-F | GTTGTCGTTGGTAGAGCCCG | 163 bp |
| qACC1-R | TGAAGGCGGAGTTTGGATGC |  |
| qDGA1-F | AACTCTGGCTGTAGCATGGC | 81 bp |
| qDGA1-R | AGTGCTGGTGTCGAGATTGC |  |
| qPAH1-F | AAAGACTGGACGCACTTGGG | 195 bp |
| qPAH1-R | CGCAGCCATCGTTCTATCGG |  |
| qERG9-F | GAAACCTGAAAACGAACAACTGG | 145 bp |
| qERG9-R | CATAACTTGGGGAATGGCACA |  |
| qOLE1-F | TTCTACTACGCTGTCGGTGG | 128 bp |
| qOLE1-R | CCTTCAACGGAAGCACAACC |  |
| qMGA2-F | TTCCTCCTGAACGGGTCTCC | 127 bp |
| qMGA2-R | CGAAAGACAACAGGTTGGGC |  |
| qIZH1-F | CTGCTGCAAGGCAACGAGAG | 153 bp |
| qIZH1-R | AGTGTTTCACGAACGTACCCG |  |
| qIZH4-F | GACGGACAAACTCATTTCCTCG | 172 bp |
| qIZH4-R | CAAAGCCACAAGTTCCCACG |  |
| qYDR274C-F | AAACGCAAGACCGAGCATTC | 94 bp |
| qYDR274C-R | CGAAACGTCCTTTTGCACGG |  |

Table S4 Down-regulated genes in strain YBX-01 after treated with oleic acid for 2 h and 12 h by transcriptome analysis

| Systematic name | Standard name | FPKM ratio* 2 h | FPKM ratio* 12 h | Description |
| --- | --- | --- | --- | --- |
| YGL055W | ***OLE1*** | 0.1594 | 0.2450 | Delta(9) fatty acid desaturase |
| YIR033W | ***MGA2*** | 0.3914 | 0.3861 | ER membrane protein involved in regulation of *OLE1* transcription |
| YDR492W | ***IZH1*** | 0.4735 | 0.4348 | Membrane protein involved in zinc ion homeostasis |
| YOL101C | ***IZH4*** | 0.1665 | 0.2831 | Membrane protein involved in zinc ion homeostasis |
| **YDR274C** | *-* | 0.1858 | 0.2246 | Putative protein of unknown function |
| YCR018C | *SRD1* | 0.4949 | 0.4563 | Protein involved in the processing of pre-rRNA to mature rRNA |
| YOL104C | *NDJ1* | 0.3148 | 0.1872 | Protein that regulates meiotic SPB cohesion and telomere clustering |
| YGR161W-B | *-* | 0.0000 | 0.0588 | Retrotransposon TYA Gag and TYB Pol genes |
| YGR259C | *-* | 0.4756 | 0.3837 | Dubious open reading frame unlikely to encode a functional protein |
| YJL136W-A | *-* | 0.0952 | 0.4976 | Putative protein of unknown function |
| YPL197C | *-* | 0.4328 | 0.3048 | Dubious open reading frame unlikely to encode a functional protein |
| YLR302C | *-* | 0.4793 | 0.1990 | Putative protein of unknown function |
| YPR076W | *-* | 0.1917 | 0.4875 | Dubious open reading frame unlikely to encode a functional protein |
| YEL053W-A | *-* | 0.0000 | 0.2792 | Dubious open reading frame unlikely to encode a functional protein |
| YOR329W-A | *-* | 0.1928 | 0.1940 | Dubious open reading frame unlikely to encode a functional protein |
| YIL068W-A | *-* | 0.4769 | 0.4520 | Dubious open reading frame unlikely to encode a functional protein |
| YGL069C | *-* | 0.0000 | 0.4717 | Dubious open reading frame unlikely to encode a functional protein |
| YER038W-A | *-* | 0.4787 | 0.0000 | Mitochondrial protein of unknown function |
| YER067C-A | *-* | 0.1881 | 0.0000 | Dubious open reading frame unlikely to encode a functional protein |
| YJR018W | *-* | 0.0000 | 0.0000 | Dubious open reading frame unlikely to encode a functional protein |
| YLL030C | *RRT7* | 0.4888 | 0.3531 | Putative protein of unknown function |

*FPKM ratio: The ratio of FPKM between oleic acid added group and the control group
